# Diffusion on social networks: Survey data from rural villages in central China

**DOI:** 10.1016/j.dib.2016.02.081

**Published:** 2016-03-09

**Authors:** Hang Xiong, Puqing Wang, Yueji Zhu

**Affiliations:** a Geary Institute for Public Policy and School of Sociology, University College Dublin, Dublin, Ireland; b College of Economics and Management, Wuhan Polytechnic University, Wuhan, China; c School of Economics and Management, Hainan University, Haikou, China

**Keywords:** High-value crop, Diffusion, Social networks, Rural China, Household survey

## Abstract

Empirical studies on social diffusions are often restricted by the access to data of diffusion and social relations on the same objects. We present a set of first-hand data that we collected in ten rural villages in central China through household surveys. The dataset contains detailed and comprehensive data of the diffusion of an innovation, the major social relationships and the household level demographic characteristics in these villages. The data have been used to study peer effects in social diffusion using simulation models, “Peer Effects and Social Network: The Case of Rural Diffusion in Central China” [1]. They can also be used to estimate spatial econometric models. Data are supplied with this article.

## 1 **Specifications Table**

**Table:** 

Subject area	Economics and sociology
More specific subject area	Agricultural Economics, Diffusion of Rural Innovations, Social Networks
Type of data	RData files (a data format generated by R), graphs
How data was acquired	Survey
Data format	Raw and analyzed
Experimental factors	Raw data are obtained from survey on all households in the survey sites. Network data are organized as weighted adjacency matrices.
Experimental features	The experiment focuses on the diffusion of innovation through social networks in rural China.
Data source location	Wuhan, China
Data accessibility	Data are provided with this article

## 2 **Value of the data**

• This dataset contains detailed diffusion data, network data and demographic data of the same survey sites. They can be used to study diffusion on social networks.
• The network data can be used to quantitatively analyze social structure of rural communities.
• The dataset can be used to demonstrate how social relationships shape economic activities.

## 3 Data

We present the latest survey data collected from 10 villages and over 400 households. This set of data includes (1) the ties of major social relationships between households (namely, network data), household level demographic characteristics (namely, demographic data), and the year in which each household adopted a new crop (namely, diffusion data).

Table 1 presents basic statistics of these villages.

**Fig. 1:**
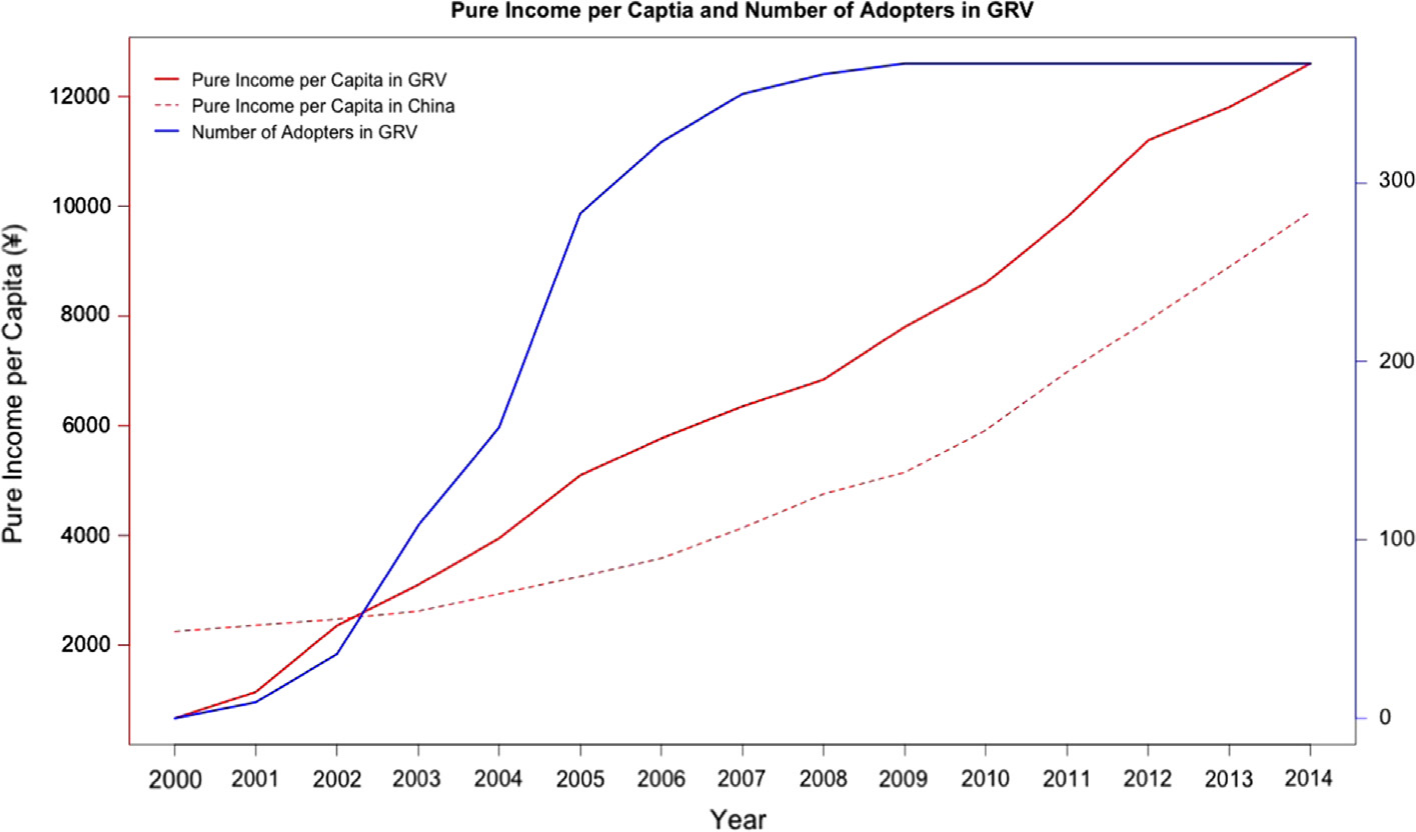
Diffusion of the new crop and change of households’ income in GRV (A high-value crop, a vegetable called *Artemisia selengensis* (AS), was introduced into the villages in 2001. By 2009, all households in these villages that were capable of planting the new crop have adopted it. As a result, the income per capita in these villages soared from less than RMB 700 in 2000 to over RMB 10,000 in 2009.).

Fig. 1 shows how the new crop was diffused in these villages (which constitute an administrative village named GRV) and how households’ income changed accordingly.

## 4 Experimental design, materials and methods

### 4.1 Sample selection

The households in each village are selected as follows. We begin with a name list of households that signed a land contract with the administrative village committee (AVC) of GRV. The numbers of households with a contract are presented in the second row of Table 3. The total number over all villages is 463. Subsequently, we excluded (1) the households that were not an independent household before signing the contracts, (2) the households whose member(s) retired from farming before 2000 – we use the remaining households to create the social networks in these villages, they thus called network sample (fifth row of Table 3) – (3) Emigrated before 2000, (4) emigrated after 2000 and had not adopted before the time they ceased to practice farming, (5) with no or limited ability to practice farming, and (6) households doing non-farming job. The remaining households constitute the sample that can be used to study the diffusion of the new crop. They thus called diffusion sample (tenth row of Table 2).

### 4.2 Network data

#### 4.2.1 Kinship data

We collected the family trees of all 63 lineages and all the intermarriages occurring between 1950 and 2000 in the ten villages. Using these data, we created the kinship ties (including blood ties and affinity ties) between the households. In addition, we calculated the weights of the ties according to the closeness in blood. We set the weight of the closest tie, i.e., the one between siblings and the one between an individual and his parents, to be 1, that of the second closest tie to be 0.5, and so forth. Refer to [2] for an elaboration on the methods of calculating weights of blood ties and weights of affinity ties.

#### 4.2.2 House neighborhood relationships

The data of house neighborhood were collected using Google Earth map, on which the houses in the villages can be clearly identified. With the aid of the tool ArcGIS, we calculated the distances between houses. We then took those within a distance smaller than 15 m as first-degree neighbors of each other and set the weight to be 1, and those houses that are located in the distance 15–30 m as the second-degree neighbors and set the weight to be 0.5.

#### 4.2.3 Land plot neighborhood relationships

The households generally have 5–8 plots of farming land. A household׳s plots are often adjacent to other households’. Plot neighborhood is an important social relationship in the villages. The information of plot neighborhood is well documented in each household׳s land contracts. We can use this information to define the plot neighborhood between households. Plots smaller than 0.5 mu is classified as small plots, those between 0.5 and 1 mu are classified as medium-sized plots, and those larger than 1 mu are large plots. According to this classification, we set the weights small, medium-sized and large plot as 1/4, 1/2 and 1, respectively.

#### 4.2.4 Political relationship data

Political relationship in the villages is composed of ties between village leaders, including AVC members and the heads of village, and party membership ties. Specifically, there are six AVC members and ten heads of village (each village has one), and they work together at least once per week. There have been 38 party members by 2014 in GRV, and they work together about once per month. We collected the names of all village leaders and party member in GRV from 2000 to 2014. To differentiate the strength of the two different kinds ties, we set the weight of a tie between village leaders to be 1, whereas the weight of a tie between party members to be 0.5.

Table 3 presents the network characteristics of the social network consisting of all types of ties in GRV.

### 4.3 Diffusion data

We conducted a questionnaire survey over all households in the villages. In the questionnaires, we asked the farmers (usually the heads of the households) the acreage of the land that they used to farm the AS in each years, especially the years in which they farmed more than 1 mu. The questionnaire results were verified by two pieces of documentary evidence: the historical imagery on the Google Earth map, from which the land plots that were planted AS in 2003 can be identified, and the record of households who received subsidies from a government program called “shopping basket” program, which recorded AS planters in 2004–2007.

### 4.4 Demographic data

We obtained detailed demographic information of each person that is registered to GRV from the Department of Residency Registration in the town and AVC. The information covers acreage of land and number of farming workers of households, and surnames, year of birth and number of schooling years of the heads of household, in each village from 2000 to 2014.

## Figures and Tables

**Table 1:** Basic statistics of surveyed villages.

**Village numbered**	**Number of households**	**Population**	**Acreage of land (mu)**
Village 1	50	270	230.04
Village 2	57	301	303.10
Village 3	35	162	117.40
Village 4	31	128	204.96
Village 5	61	276	317.18
Village 6	67	336	148.80
Village 7	59	272	303.65
Village 8	19	87	124.09
Village 9	57	269	467.82
Village 10	27	127	221.83
Total	463	2228	2438.87

**Table 2:** Selection of sample households.

# of Households	Village											
1	2	3	4	5	6	7	8	9	10	Total	Percentage (**%)**
***Households with contract***	**50**	**57**	**35**	**31**	**61**	**67**	**59**	**19**	**57**	**27**	**463**	**100**
Non-independent households	1	0	2	0	0	2	2	0	1	2	10	2.16
Non-farming parent(s) households	0	3	5	4	5	3	4	1	0	2	27	5.83
***Households in network sample***	**49**	**54**	**28**	**27**	**56**	**62**	**53**	**18**	**56**	**23**	**426**	**92.01**
Households emigrating before 2000	5	2	1	1	4	1	0	0	1	1	16	3.46
Emigrating after 2000 and non-adopting	1	1	2	1	3	2	2	1	2	0	15	3.24
Households lack of labor	0	4	0	1	5	3	6	0	3	1	23	4.97
Non-farming households	1	1	1	0	0	0	2	0	0	0	5	1.08
***Households in diffusion sample***	**43**	**46**	**26**	**24**	**44**	**58**	**45**	**17**	**51**	**23**	**367**	**79.27**

**Table 3:** Network characteristics.

**Matric**	**Mean**	**Std. dev.**	**Matric**	**Mean**	**Std. dev.**
Average degree	26.77	11.03	Diameter	2.18	0.47
Strength	6.43	1.10	Degree centralization	0.54	0.15
Density	0.68	0.19	Closeness centralization	0.005	0.002
Clustering coefficient	0.83	0.13	Betweenness centralization	0.08	0.03
Average path length	1.08	0.24	Small-worldness	−0.24	0.08
