# Supplementary figures and images for: Diffusion on social networks: Survey data from rural villages in central China
Source: Data Brief. 2016 Mar 9;7:546–50. doi: 10.1016/j.dib.2016.02.081 (PMC4802424; doi:10.1016/j.dib.2016.02.081)

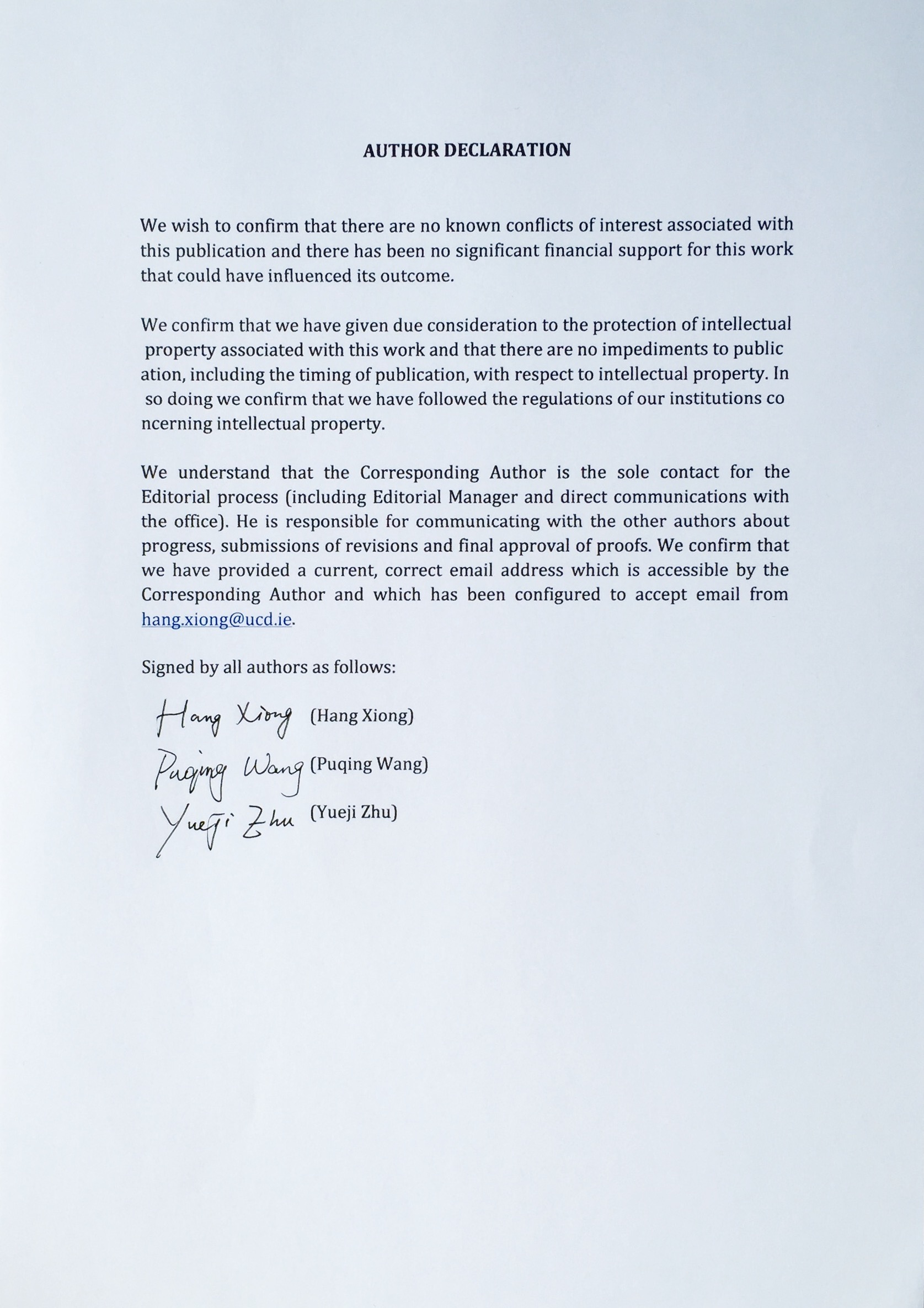

Supplement: Supplementary file 1 — Supplementary material [file mmc1.zip › Author decaration.jpg]
